# The impact of Community Mobilisation on HIV Prevention in Middle and Low Income Countries: A Systematic Review and Critique

**DOI:** 10.1007/s10461-014-0748-5

**Published:** 2014-03-23

**Authors:** Flora Cornish, Jacqueline Priego-Hernandez, Catherine Campbell, Gitau Mburu, Susie McLean

**Affiliations:** 1 Department of Methodology, The London School of Economics and Political Science, London, UK; 2 Department of Social Psychology, The London School of Economics and Political Science, 3rd Floor St Clements Building, Houghton Street, London, WC2A 2AE UK; 3 International HIV/AIDS Alliance, Brighton, UK; 4 Division of Health Research, Lancaster University, Lancaster, UK

**Keywords:** Community mobilisation, Community participation, HIV prevention, HIV/AIDS, Systematic review

## Abstract

While community mobilisation (CM) is increasingly advocated for HIV prevention, its impact on measurable outcomes has not been established. We performed a systematic review of the impact of CM within HIV prevention interventions (N = 20), on biomedical, behavioural and social outcomes. Among most at risk groups (particularly sex workers), the evidence is somewhat consistent, indicating a tendency for positive impact, with stronger results for behavioural and social outcomes than for biomedical ones. Among youth and general communities, the evidence remains inconclusive. Success appears to be enhanced by engaging groups with a strong collective identity and by simultaneously addressing the socio-political context. We suggest that the inconclusiveness of the findings reflects problems with the evidence, rather than indicating that CM is ineffective. We discuss weaknesses in the operationalization of CM, neglect of social context, and incompatibility between context-specific CM processes and the aspiration of review methodologies to provide simple, context-transcending answers.

**Electronic supplementary material:**

The online version of this article (doi:10.1007/s10461-014-0748-5) contains supplementary material, which is available to authorized users.

## Resumen

Mientras que la movilización de la comunidad (MC) es cada vez más recomendada para la prevención del VIH, su impacto en resultados mensurables no se ha establecido. Realizamos una revisión sistemática del impacto de la MC en intervenciones para la prevención del VIH (N = 20), en resultados biomédicos, conductuales y sociales. En grupos de riesgo (particularmente trabajadoras sexuales) la evidencia es más bien consistente, indicando una tendencia general de impacto positivo, siendo los resultados conductuales y sociales más robustos que los biomédicos. En los jóvenes y comunidades en general la evidencia no es concluyente. Resultados favorables parecen ser mejorados al involucrar grupos con una fuerte identidad colectiva y, simultáneamente, atender al contexto socio-político. Proponemos que la naturaleza poco concluyente de los hallazgos refleja problemas con la evidencia, en lugar de sugerir que la MC es inefectiva. Discutimos las debilidades en la operacionalización de la MC, la desatención al contexto social y la incompatibilidad entre procesos contextualmente específicos en la MC y la pretensión de las metodologías de revisión de proveer respuestas simples y que trasciendan el contexto.

## Introduction

Many HIV intervention programmes have disappointing outcomes, often ascribed to a lack of community ‘buy-in’. It is often the case that outsiders such as academics, multilateral agencies, or international organisations implement interventions without assessing their relevance to their particular target community. Social research has documented failures of HIV interventions to resonate with local norms, cultures and needs [1, 2]. Behaviours advocated by external health professionals may not be feasible in contexts of poverty, political conflict and gender inequalities [2, 3]. As a result, there is a growing emphasis on the need for community involvement in the planning, implementation and ownership of interventions. Indeed, community mobilisation (CM) is now widely considered a “critical enabler” of an effective HIV/AIDS response [4]. Despite this increasing interest in CM, there has been little systematic attention to its impacts.

One reason for the lack of systematic attention to CM is that it is used across categories of HIV intervention usually considered separately, namely, biomedical, behavioural and structural interventions. CM has been argued to be valuable in recruiting men to take up circumcision in biomedical interventions [5, 6]. Similarly, in behavioural interventions, CM may be used to recruit participants through outreach, or to inform culturally-appropriate materials [7, 8]. CM is often termed a ‘structural intervention’ when it is considered as a means of empowering marginalised communities and thus changing power relations [9–12]. For a smaller number of studies, CM is considered their main mechanism of intervention [13–16]. Although the impacts of CM have not yet been subject to a systematic review, such a review stands to contribute to research and practice across the range of HIV prevention approaches, wherever CM might be considered a ‘critical enabler’. The term ‘community mobilisation’ is not consistently defined, theorised, operationalized or systematically appraised [17]. In its fullest operationalization, CM seeks “to create and harness the agency of the marginalised groups most vulnerable to HIV/AIDS, enabling them to build a collective, community response, through their full participation in the design, implementation and leadership of health programmes and by forging supportive partnerships with significant groups both inside and outside of the community” [18]. This definition sets a high bar, and many operationalizations of CM are far less ambitious [19]. Although our definition preserves the conceptual distinctiveness of CM, we aimed to be relatively inclusive in this review, for very few published evaluations implement the ‘maximalist’ version of CM as defined above.

For the purposes of this review, we take the term ‘community’ to refer to collective resources that exist among a community, rather than at the individual level. We take the term ‘mobilisation’ to mean capitalising on those community connections and strengths to generate new possibilities of action. In keeping with the ‘minimalist’ definition of CM, in this paper we consider CM as a component of externally-triggered HIV interventions, rather than including indigenous CM initiated by grassroots actors with broader interests than HIV. This latter topic is addressed in the literature on social capital and HIV/AIDS [20–22].

Research on CM to date has tended to be qualitative, grounded in ethnographic methods, and to focus on processes rather than on outcome evaluation [13, 23–25]. A growing body of social research has underscored the role of CM in building HIV competent communities, ensuring interventions are relevant and accessible to local people, and enabling people to work collectively to create health-enabling environments [18, 26, 27]. This work has also emphasised the significance of partnerships between communities and outside agencies as a key supportive condition for effective CM [18, 28, 29]. Furthermore, the pertinence of Western conceptualisations of CM and its constituting elements needs to be validated in specific intervention contexts [17]. Such information is crucial to the appropriate design and evaluation of CM programmes. In the context of the ascendant ‘evidence-based policy and practice’ movements, however, quantitative evaluations, and systematic reviews of these, are valued as sources for informing policy or funding decisions.

The first aim of the current article, therefore, is to present a systematic review of studies of the impacts of CM as a component of complex HIV prevention interventions. The scope of this review is comprehensive in that we do not restrict it to any target group, and we consider the impact on biomedical, behavioural, and social outcome variables. The review draws conclusions about whether CM ‘works’ or not, and delineates more nuanced lessons about the conditions under which CM is more likely to succeed.

A systematic appraisal of the CM intervention literature is challenging. Mobilisation efforts are referred to by many terms (e.g. community solidarity, social mobilisation, community participation, community engagement), which defy simple search strategies. In addition, CM almost invariably constitutes ‘complex interventions’ [30], entailing multiple, indirect pathways between intervention and outcomes. A wide and unresolved debate surrounds the best way to deploy and evaluate CM interventions (CMI) [31, 32], with many arguing that the complex and improvisational nature of good CM defies summary in the linear ‘input–output’ models of change that characterise the ‘gold standard’ approach of randomised controlled trials (RCT). The second aim of the article is to reflect on the methodological challenges of operationalizing, evaluating and reviewing CMI.

## Methods

The definition of CM cited above sets a high standard. However, many studies which use the term ‘CM’ do so in a very limited way, for instance using ‘CM’ to simply mean reaching out the community for service or research recruitment. Heeding this concession, our key criterion was that CMI should seek to foster new capacities in a community by facilitating meaningful contact among community members. The reviewed studies aimed to engage communities in one or more of the following: enhancing supportive interpersonal relationships, building within-community support and solidarity (bonding social capital), and building bridges between communities and outside support partners (bridging social capital). In order to do so, interventions employed activities such as setting-up peer support groups and clubs, fostering of community-based organisations, performing dramas, rallies and awareness camps, creating community centres as ‘safe spaces’ for debate and conscientisation, as well as holding multi stake-holder meetings and advocacy. On the methodological front, we included reports on the impact of CM as a component of more complex interventions, and excluded articles where the impact of a singled-out mobilisation activity (e.g. peer group membership) was measured.

The following *questions* guided the review:

- To what extent does CM impact on measurable HIV-related prevention outcomes?
- Is there a significant relationship between the implementation of programmes with a CM component and biomedical, behavioural and social outcomes? If so, what is the direction of this relationship for each outcome?
- Under what programmatic conditions (target population and intervention components) is CM most successful?
- What are the methodological challenges of evaluating and synthesising evidence on CM?

### Selection

**Fig. 1:**
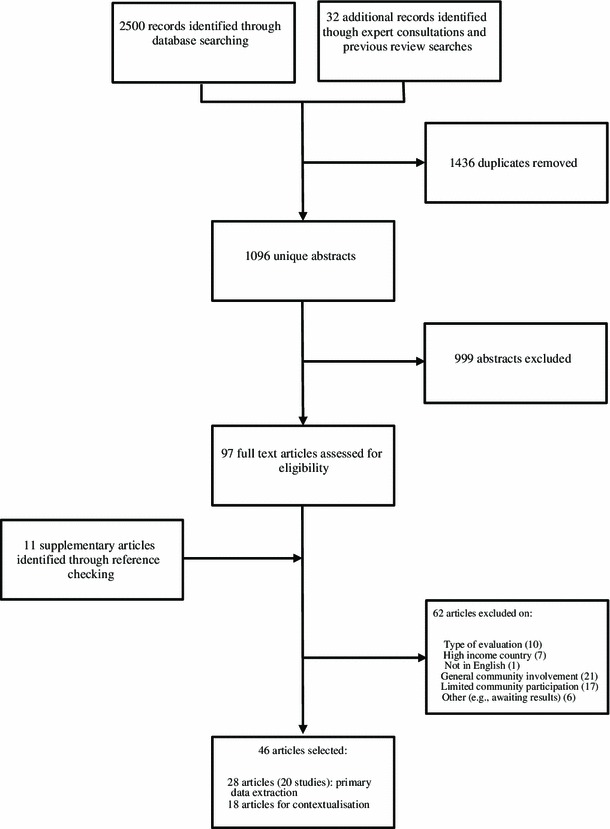
Flow of information chart

Standard systematic review procedures were followed (Fig. 1). The bibliographic databases SCOPUS, PubMed, Cumulative Index to Nursing and Allied Health Literature and PsycInfo were interrogated using free-text terms to produce a sensitive search, adjusting terms depending on the search tools available (e.g. truncation). Searches included a combination of the following terms: “intervention” AND “hiv OR aids” AND “community mobili*” OR “community particip*” OR “community led” OR “community based” OR “community activit*” OR “community development” OR “capacity building” (full search strings by database in supplementary online information). When possible, irrelevant publication types (e.g. commentaries) were excluded using search tools. A complementary search was carried out through expert consultation and systematic reference screening of previous related reviews [12, 33, 34], which rendered 32 records.

Records identified were systematised and, after removing duplicates, 1096 abstracts were screened. Of these, 97 were selected for full-text retrieval. To be included, articles needed to have been published in English in the last 11 years (from January 2003 to October 2013), and to report studies conducted in low-income, lower-middle-income or upper-middle-income economies [35]. Given that the nature of HIV epidemics, and understandings of CM have changed greatly historically, the timespan was chosen to reflect contemporary research and practice. We used three main inclusion criteria, including studies which:

- Reported community-based initiatives (as opposed to health facility-based medical interventions) that engaged one or more community groups in concrete participatory activities. *Studies should have reported modes of CM that foster meaningful community connections.*
- Evaluated the intervention in terms of at least one quantifiable biomedical (incidence and/or prevalence of HIV-1, HSV-2 and bacterial STI) or behavioural (reported condom use, reported health-service use, and HIV test-taking) outcome.
- Evaluated outcomes with reference to a comparator or control, irrespective of research design.

The second inclusion criterion is based on the rationale that CM, and derived social outcomes, shapes sexual behaviours, which in turn impact on biomedical indicators. Thus, we targeted the proximal outcomes of this logic model. Although reported condom use has been found to be poorly associated with biomedical markers [36–38], we included condom use as a behavioural outcome because it is a proxy measure of actual sexual risk historically applied in the prevention literature, affording some degree of comparability across studies. Behavioural outcomes were expanded *ad*-*hoc* to engagement in extramarital sex for one study [39], on the assumption that this might increase a person’s HIV risk [40]. This study was selected given its careful consideration of community involvement.

To enable the implementation of the selection criteria, and given the diversity of terminology employed, two steps were taken in the selection process. First, during abstract screening, records reporting the same study were clustered together, so as to gather as much information about a study as possible. Second, during full-text vetting, “reference list checking” [41] was performed to aid final selection. Eleven articles were selected from this search and assessed for inclusion.

### Data Extraction and Synthesis

Twenty unique studies were identified, documented by 28 primary and 18 supplementary articles. When possible, the unit of analysis consisted of studies rather than articles. Bibliographic data were noted for each study, as were methodological details such as sample, control or comparison and intervention components (Table 1). Outcomes identified in each study were classified into biomedical, behavioural and social. Biomedical outcomes reported in included articles addressed incidence and/or prevalence of HIV-1, HSV-2 and bacterial STI. Behavioural outcomes were limited to reported condom use, reported health-service use, and HIV test-taking. Social outcomes, such as collective efficacy or community cohesion, were included only if in a study that also reported at least one biomedical or behavioural outcome. Social outcomes considered any measurement of collectivisation, cohesion, partner violence and/or participation and excluded individualised accounts of personal development, individual empowerment or autonomy. None of the studies reported structural outcomes such as changes in legislation or policy implementation.

**Table 1:** Analytical table

Reference	Intervention name (if any) and setting	Study design^a^ and length	Target group, sample size at baseline	Comparator	Intervention components	Outcome/results^b^		
						Biomedical	Behavioural	Social
Doyle et al. [50]	MEMA kwa Vijana 10 rural communities in Mwanza, rural Tanzania	RCT Average exposure 5.4 years prior survey	Adolescents aged 14 years or more at programme onset. N = 13814 (males: nI = 3807, nC = 3493; females: nI = 3276, nC = 3238)^c^	10 randomised comparison communities with no intervention	Participatory school programme. Training and provision of health workers for youth-friendly SRH services. Community-based condom social marketing by youth. Initial community mobilisation.	No difference between intervention and control groups in HIV prevalence (males aPR 0.91, 95 % CI 0.50–1.65; females aPR 1.07, 95 % CI 0.68–1.67) or in HSV-2 (males aPR 0.94, 95 % CI 0.77–1.15; females aPR 0.96, 95 % CI 0.87–1.06). No difference in prevalence of active syphilis (males aPR 1.1, 95 % CI 0.72–1.72; females aPR 0.91, 95 % CI 0.65–1.28), chlamydia (males aPR 1.24, 95 % CI 0.66–2.33; females aPR 1.27, 95 % CI 0.87–1.86), and gonorrhoea (males aPR 0.71, 95 % CI 0.21–2.41; females aPR 0.73, 95 % CI 0.20–2.63).	Reported condom use at last sex with non-regular partner increased among females (aPR 1.34, 95 % CI 1.07–1.69) with no difference among males (aPR 1.15, 95 % CI 0.97–1.36). No significant differences in reported condom use at last sex (males aPR 1.19, 95 % CI 0.91–1.54; females aPR1.27, 95 % CI 0.97–1.67).	NA
Kamali et al. [54]	Masaka district, Uganda 12 communities (groups A and B)	RCT Median follow-up of 3.6 years.	All adults (13 years or older). nA = 4931 nB = 4787, nC = 4836	6 communities received routine government health facilities with general community development activities (group C, control)	Group A. IEC intervention: knowledge acquisition, skill development, motivational support and attitudes development. Community-level: group meetings, one-to-one discussions, information leaflets, local drama, videos. Group B. A + STI intervention: trained health workers in STI syndromic management. Consistent supply of drugs and supervision, coupled with community-based STI health education. Group C (control). Community development and general health-related issues: self-support groups and clubs, home-based care, and general health promotion seminars.	No difference between intervention (A/B) and control (C) groups in HIV-1 incidence (A *vs* C, aIRR 0.94, 95 % CI 0.60–1.45, *p* = 0.72; B *vs* C, aIRR 1.00, 95 % CI 0.63–1.58, *p* = 0.98). Lower HSV2 incidence in group A than in group C (aIRR 0.65, 95 % CI 0.53–0.80, *p* = 0.003); no difference between group B and C (aIRR 1.00, 95 % CI 0.54–1.85, *p* = 0.99). No difference between group A and group C in active syphilis (aIRR 1.02, 95 % CI 0.66–1.57, *p* = 0.92) and high titre active syphilis (aIRR 1.03, 95 % CI 0.59–1.79, *p* = 0.90). Lower incidence in group B than in group C of active syphilis (aIRR 0.77, 95 % CI 0.61–0.96, *p* = 0.029) and high titre active syphilis (aIRR 0.52, 95 % CI 0.27–0.98, *p* = 0.044). Lower gonorrhoea prevalence in group B than in group C (aPR 0.25, 0.10–0.64, *p* = 0.013), but no difference between group A and group C (aPR 0.64, 95 % CI 0.25–1.59, *p* = 0.26). No difference between intervention and control groups for chlamydia prevalence.	No differences in condom use with last partner in group A compared with C (aPR = 1.12, 95 % CI 0.99-1.25, *p* = 0.57). Increase in condom use with last casual partner in group B compared with C (aPR = 1.27, 95 % CI 1.02–1.56, *p* = 0.036). No difference in reported ever condom use (A *vs* C, aPR = 0.92, 95 % CI 0.68–1.26, *p* = 0.54; B *vs* C, aPR = 1.10, 95 % CI 0.95–1.26, *p* = 0.16).	NA
Cowan et al. [49]	Regai Dzive Shiri 15 communities in rural Zimbabwe	RCT 4 years	18–22 year-olds Intervention = 3381 Control = 3410	15 randomised control villages with delayed intervention. Matched controls for each cohort	Participatory youth programme via peer educators. Programme for parents and community stakeholders aimed at enhancing knowledge, communication and community support towards adolescents. Training for nurses and other clinic staff.	No difference between intervention and control groups in HIV or HSV-2 prevalence in men (aOR 1.20, 95 % CI 0.66–2.18; aOR 1.23, 95 % CI 0.69–2.18) and women (aOR 1.15, 95 % CI 0.81–1.54; aOR 1.24, 95 % CI 0.93–1.65).	No difference between intervention and control groups in reported condom use at last sex in men (aOR 1.03, 95 % CI 0.83–1.29) and women (aOR 0.93, 95 % CI 0.72–1.20). No difference in clinic attendance in men (aOR 0.99, 95 % CI 0.76–1.29) and women (aOR 0.98, 95 % CI 0.76–1.28).	NA
Gregson et al. [52]	Manicaland 6 communities in Manicaland, Zimbabwe	RCT 3 years	Males (17–54 years) and females (15–44 years) Intervention = 4792 Control = 4662	6 matched control communities with standard government services	Peer education and condom distribution: sex workers, male clients and general community. Income-generating projects (withdrawn). Strengthened syndromic management of STI. Open days with HIV/AIDS IEC activities.	No difference between intervention communities and control communities in HIV incidence (aIRR 1.27, 95 % CI 0.92–1.75, *p* = 0.12).	Reported unprotected sex with casual partners higher in intervention than in control communities (males aPOR 1.46, CI 1.02–2.09, *p* = 0.039; females aPOR 6.51, CI 2.14–19.82, *p* = 0.001). No group differences in consistent condom use with regular partners (males aPOR 1.01, CI 0.72–1.40, *p* = 0.975; females aPOR 1.09, CI 0.72–1.65, *p* = 0.686) or treatment-seeking within 3 days of STI symptoms (males aPOR 1.13, CI 0.59–2.16, *p* = 0.709; females aPOR 1.14, CI 0.74–1.77, *p* = 0.544).	NA
Jewkes et al. [51]	Stepping stones	RCT 6 to 8 weeks	N = 2,776 Young men (nI = 694, nC = 666) and women (nI = 715, nC = 701) aged 15 to 26 years	Control intervention: single three-hour session on HIV, safer sex and condoms. Baseline and follow-up 12 and 24 months later	Preliminary community consultation. Participatory learning approaches: critical reflection, role play and drama. 13 three-hour long sessions. 3 peer group meetings. 1 final community meeting.	No difference in HIV incidence among men and women (aIRR 0.95, 95 % CI 0.67–1.35, *p* = 0.78) HSV2 incidence lower in the intervention than the control group (aIRR 0.67, 95 % CI 0.46–0.97, *p* = 0.036)	No difference between intervention and control groups in correct condom use at last sex at 12 and 24 months in men (aOR 1.26, 95 % CI 0.92–1.74, *p* = 0.16; aOR 0.88, 95 % CI 0.64–1.21, *p* = 0.43) and women (aOR 0.95, 95 % CI 0.72–1.28 *p* = 0.79; aOR 0.90, 95 % CI 0.70–1.17 *p* = 0.45).	Men reported perpetration of intimate partner violence less frequently than controls at 12 months and 24 months (aOR 0.73, 95 % CI 0.50–1.06 *p* = 0.099; aOR 0.62, 95 % CI 0.38–1.01, *p* = 0.054), with no difference among women (aOR 0.87, 95 % CI = 0.64–1.18, *p* = 0.36; aOR 1.14, 95 % CI = 0.77–1.68, *p* = 0.51)
Pronyk et al. [53]	IMAGE 4 villages in Limpopo, South Africa	RCT 18 months	Three cohorts: 1. Women who applied for loan (nI = 426, nC = 417; 2. 14–35 years old household co-residents (nI = 725, nC = 730); 3.Randomly selected community members (nI = 746; nC = 736)	4 matched villages with delayed intervention. Matched controls for each cohort	Poverty-focused microfinance. Gender and HIV participatory curriculum (structured training and community mobilisation). Community mobilisation as HIV and intimate partner violence awareness, village workshops and multi stake-holder meetings, marches, partnerships and committees.	No difference between cohort 3 and control group in HIV incidence (aRR 1.06, 95 % CI 0.66–1.69).	No difference in unprotected sexual intercourse with a non-spousal partner in cohort 2 (aRR 1.02, 95 % CI 0.85–1.23) and cohort 3 (aRR 0.89, 95 % CI 0.66–1.19) relative to controls. No difference in having had an HIV test in cohort 2 (aRR 1.18, 95 % CI 0.73–1.91) and cohort 3 (aRR 1.09, 95 % CI 0.81–1.47) relative to controls.	Intimate partner violence reported less frequently in cohort 1 than in control group (aRR 0.45, 95 % CI 0.23–0.91). Cohort 1 reported higher levels of participation in social groups (aRR 1.85, 95 % CI 0·95–3·61) and collective action (aRR 2·06, 95 % CI 0.92–4.49) than control group. No difference in sense of community solidarity in times of crisis(aRR 1.65, 95 % CI 0.81–3.37) and belief of community work on common goals (aRR 1.11, 95 % CI 0.38–3.24).
Sweat et al. [15]	Accept, HPTN 043 Communities of Tanzania (10), Zimbabwe (8) and Thailand (14)	RCT 3 years	16–32 year-olds Clients: Tanz (nI = 6250; nC = 6733); Zimb (nI = 10,700; nC = 12,150); Thai (nI = 11,290; nC = 10,033)	Equal number of matching communities received standard clinic-based voluntary counselling and testing (SVCT) for HIV	Preliminary participatory mapping. Community mobilisation: community working groups, outreach workers activities, community-based outreach workers. Community based voluntary counselling and testing (CBVCT): accessible mobile VCT for and community based-support services after testing.	NA	Percentage of “clients” testing for HIV for the first time higher in CBVCT communities than in SVCT communities. Crude mean difference: 40.2 % (95 % CI 15.8–64.7, *p* = 0.019) across one pair of matched communities per country.	NA
Coates et al. [48]	Accept, HPTN 043					No difference HIV incidence in CBVCT than in SVCT (RRi 0.86, 95 % CI 0.73–1.02, *p* = 0.08).	NA	NA
Kerrigan et al. [14]	Santo Domingo and Puerto Plata in Dominican Republic	Cohort analytic 1 year	68 FSW establishments in 2 cities N ~ 200	Baseline and follow-up. Components 1–4 (CM) in both cities, component 5 (policy) only in Puerto Plata. Exposure to intervention *vs* low-exposure	1. Solidarity and collective commitment. 2. Environmental cues. 3. Clinical services. 4. Monitoring and encouraging adherence. 5. Policy and regulation.	Prevalence of 1 or more STIs (gonorrhoea, trichomoniasis, or chlamydia) decreased in Puerto Plata (aOR 0.50, 95 % CI 0.32–0.78, *p* < 0.01) but had no significant changes in Santo Domingo (aOR 0.60, 95 % CI 0.35–1.03).	Consistent condom use with new clients increased in Santo Domingo, (aOR 4.21, 95 % CI 1.55–11.43, *p* < 0.01) but not in Puerto Plata (aOR 2.27, 95 % CI 0.47–10.84). Consistent condom use with regular partners and FSW’s *observed* verbal rejection of unsafe commercial sex increased in Puerto Plata (aOR 2.97, 95 % CI 1.33–6.66, *p* < 0.01; aOR 3.86, 95 % CI 1.96–7.58, *p* < 0.001) but not in Santo Domingo (aOR 1.29, 95 % CI 0.62–2.70; aOR 1.49, 95 % CI 0.81–2.73).	NA
Gao and Wang [72]	Chengdu, Southern China	Cohort analytic 5 months	MSM N = 160 Intervention n = 80; Control n = 80	Baseline and follow-up. Control received no exposure to intervention	Gay bar-based participatory entertainment-education. Outdoor edutainment activities to expand networks. Fostering of a caring environment.	NA	Increase in condom use among intervention group with casual partners: vaginal sex (6.1–73.5 %, *p* < 0.001), anal sex (4.3–76.8 %, *p* < 0.001) and oral sex (1.4–15.9 %, *p* < 0.01); with regular partners: vaginal sex (7.4–40.7 %, *p* < 0.001), anal sex (3.1–46.2 %, *p* < 0.001) and oral sex (1.5–10.3 %, *p* < 0.05). No differences in condom use among comparison groups.	NA
Swendeman et al. [65]	Sonagachi-replication West Bengal, India	Cohort analytic 16 months	FSW, 1 replication community N = 216	Baseline and 3 follow-ups within 16 months. Control community receiving standard care: STD clinic free of charge, condom promotion and peer education	Enhanced intervention including standard care plus: Rapid appraisal. STD/HIV Intervention Project. FSW community organisation. USHA multi-purpose micro-finance cooperative. Advocacy with stake-holders and power-brokers.	NA		Social support through organising and solidarity increased among intervention group (*p* < 0.001 across items), while it decreased among controls (*p* < 0.001 across items). Political participation remained stable among intervention group (*p* > 0.05) and increased among controls (*p* < 0.001)
Basu et al. [66]			Intervention n = 110; control n = 106				Condom use increased more among FSW in intervention community than among controls (B = 0.3447, *p* = 0.002).	
Erausquin et al. [56]	Parivartan Rajahmundry, Andhra Pradesh, India	Cohort analytic 6 years: Project onset in 2004, 3 surveys: 2006, 2007, 2009/10.	FSW N = 812	3 time points. Comparison between no programme exposure, receptive exposure and active utilisation	Identification of social change agents among FSW. Peer education and community organisation. Traditional intervention (condom distribution, condom use promotion, etc.). Advocacy and organisation of FSW-led CBOs.	NA	Greater programme exposure related to increased probability of consistent condom use with clients (no exposure *vs* receptive exposure aOR 1.57, 95 % CI 1.22–2.02, *p* < 0.001; no exposure *vs* active utilisation aOR 2.03, 95 % CI 1.60–2.57, *p* < 0.001). Association maintained over time.	NA
Blankenship et al. [55]	Parivartan	Case control 2 years (NA - see comparison)	N = 812	Comparison between no programme exposure, receptive exposure and active utilisation		NA	Greater programme exposure related to increased probability of consistent condom use (no exposure *vs* active utilisation aOR 2.09, 95 % CI 1.48–2.94, *p* < 0.005).	Greater programme exposure associated with increased collective identity, collective efficacy and collective agency (no exposure *vs* active utilisation *p* < 0.001 in bivariate analysis for all items).
Lippman et al. [67]	Encontros Corumba, Brazil	Cohort Analytic Baseline and follow-up at 3, 6, 9 and 12 months later.	Sex workers: female, male and transvestite. N = 420	Baseline and 4 follow-ups. Ever exposed *vs* unexposed	Individual and interpersonal level: increased health services provision, counselling, free condoms provision, peer education and outreach. Community level: forging of community partnerships, collective activities (e.g. workshops), mobilisation for dialogue, formation of a sex workers association, distribution of destigmatising materials.	No differences in probability of incident STI for exposed participants compared with unexposed (aOR 0.46, 95 % CI 0.2–1.3).	Exposure related to higher likelihood of reporting consistent condom use with regular clients (aOR 1.9, 95 % CI 1.1–3.3, *p* < 0.05), with no differences with new clients (aOR 1.6, 95 % CI 0.9–2.8) and non-paying partners (aOR 1.5, 95 % CI 0.9–1.5).	Active participants increased participation in networks (SD 0.3, 95 % CI 0.1–0.5) and did not change perceived cohesion (SD 0.1, 95 % CI −0.1 to 0.24).
Gutierrez et al. [63]	Frontiers Prevention Project Andhra Pradesh, India	Cohort analytic Baseline and follow-up 4 years later.	FSW & MSM in 12 geographically distinct sites N = 2786MSM (1680 FPP, 1106 non-FPP); 3442 FSW (1692 FPP, 1750 non-FPP).	Baseline and follow-up. Non-FPP (12 sites) receiving Avahan intervention	STI services, behaviour change communication, condom programmes, community mobilisation, and enabling and structural interventions. Emphasis on social capital building, network and support formation, empowerment, violence reduction, referrals for HIV testing and basic AIDS care services.	Intervention correlated with lower probability of sero-positivity to HSV-2 and syphilis among both MSM (*p* < 0.001, *p* < 0.05) and FSW (*p* < 0.05, *p* < 0.001).	MSM: intervention related to increase in condom use with last female sexual partner (*p* < 0.05) but no difference with last male sexual partner. FSW: intervention associated with increase in condom use with regular partners (*p* < 0.05) but no difference with last sexual client.	NA
Gutierrez et al. [69]	Frontiers Prevention Project Ecuador	Cohort analytic Baseline and follow-up 4 years later.	FSW & MSM in 6 cities N = 1727 MSM (1248 FPP, 479 non-FPP); 1526 FSW (752 FPP, 774 non-FPP)	Baseline and follow-up. 3 intervention cities *vs* 3 comparison cities with standard national HIV initiatives	Individually focused health promotion. Ensuring access, scaling-up targeting and improving service and commodity delivery. Mobilisation of key populations’ communities (participatory assessment, leadership workshops, human rights promotion, establishing of solid networks, safe meeting spaces provision). Advocacy, policy change and community awareness. Capacity building of NGOs and CBOs to effectively implement quality prevention interventions.	MSM: No difference in seroprevalence of HIV (aOR 0.84, 95 % CI 0.31 − 2.31) and HSV-2 (aOR 0.40, 95 % CI 0.11–1.5). Reduced odds of syphilis seroprevalence (aOR 0.32, 95 % CI 0.14–0.72). FSW: No difference in seroprevalence of HIV (aRR 0.75, 95 % CI 0.42–1.3) and syphilis (aRR 1.08, 95 % CI 0.70–1.67). Lower risk of HSV2 seroprevalence (aRR 0.93, 95 % CI 0.85–0.99).	MSM: Higher odds of condom use with a male partner associated with intervention (aOR 2.89, 95 % CI 1.34–6.28). No difference in condom use with female partners (aOR 1.91, 95 % CI 0.47–7.8). FSW: No difference in condom use with clients (aRR 0.98, 95 % CI 0.93–1.00) and regular partners (aRR 1.29, 95 % CI 0.85–1.90) associated with intervention.	NA
Kerrigan et al. [70]	Sonagachi-inspired 3 sites in Rio de Janeiro, Brazil	Cohort 18 months	FSW older than 18 years N = 499	Pre-post intervention	Current FSW peer educator as agents of social change, trained to bring about and discuss issues of common concern. FSW organisation. Drop-in centre as safe space to discuss and hold project workshops and activities. Community identified priority action areas that received funding and technical assistance.	NA	No difference in consistent condom use with all clients in the last 4 months (87.2–88.6 %, *p* = 0.287) and consistent condom use with all (paying and non-paying) partners in last week (80.40–79.0 %, *p* = 0.808).	Social participation increased from 0.74 to 1.23 (*p* < 0.0001). No differences in sense of community as social cohesion and mutual aid (values not reported).
Williams et al. [71]	Carletonville project. Mothusimpilo intervention. Carletonville, South Africa.	Cohort 2 years.	Miners and sex workers, general population. Miners = 899 Sex workers = 121 Men = 443 Women = 691	Baseline and follow-up	Community-based peer education, condom distribution, syndromic management of STIs and presumptive STI treatment for sex workers.	Increases in prevalence of: Syphilis: among miners (5.5–8.3 %, aOR 1.57, *p* = 0.02) and women (9.8–18.7 %, aOR 2.06, *p* < 0.01). Gonorrhoea: miners (3.0–7.4 %, aOR 2.61, *p* = 0.01). Chlamydial infection: miners (3.8–13.9 %, aOR 4.23, *p* < 0.01), men (3.6–12.4 %, aOR 3.54, *p* < 0.01) and women (7.9–13.8 %, aOR 1.88, *p* < 0.01). No differences in STI were computed for sex workers in syphilis (25.0– 34.4 %, aOR 1.56, *p* = 0.15)., gonorrhoea (15.7–16.1 %, aOR 1.01, *p* = 0.96) and chlamydial infection (9.1–12.9 %, aOR 1.45, *p* = 0.40).	“Ever used a condom” report increased among miners (39.5–51.3 %, aOR 1.66, *p* < 0.01) and women (33.1–42.3 %, aOR 1.58, *p* < 0.01), but not among men (48.1–54.8 %, aOR 1.23, *p* = 0.17). “Always use condoms with casual partner” report increased among miners (13.2–27.2 %, aOR 2.45, *p* < 0.01) men (14.7–35.6 %, aOR 3.19, *p* < 0.01) and women (17.8–24.9 %, aOR 1.56, *p* = 0.03). No significant differences were reported among sex workers for “ever used a condom” (69.7–77.2 %, aOR 1.39, *p* = 0.34) and “always use condoms with casual partner” (54.3–41.9 %, aOR 0.57, *p* = 0.07).	NA
Schensul et al. [39]	RISHTA 3 communities of Mumbai, India	Cohort 3 years	Married men aged 21–40 yrs. N = 2408	Pre-post intervention. Longitudinal panel sample (n = 403)	Formative community mapping. Community education: street dramas, community meetings, poster sessions, banner presentations, videos/movies, printed materials, interpersonal communication.	NA	Change in extramarital sex related to change in alcohol use (*p* < 0.01). Men who were drinkers in BL but non-drinkers in EL and non-drinkers more likely to report reduction in extramarital sex compared to their drinkers counterparts.	NA
Benzaken et al. [68]	Princesinha Project in Manacapuru, Amazonas State, Brazil	Cohort 2 years	FSW N = 148	Baseline and follow-up	Sex workers peer-education and referrals to services. Activities on a daily and weekly basis. Activities to increase project visibility and to foster sex workers’ social inclusion. Peer educators conducted mapping of condom retail locations and data collection about the town’s sex work networks and dynamics.	NA	Increases in reported condom use for oral sex with clients (37.2– 56.1 %, *p* < 0.001), in all situations (0.0–77.7 %, *p* < 0.001) and during last week (41.9–78.0 %, *p* < 0.001). Non-significant increases in reported condom use in anal sex with clients (37.2–48.2 %, *p* = 0.050) and in vaginal sex with clients 68.9–77.7 %, *p* = 0.090).	NA
Guha et al. [59]	Avahan States of Tamil Nadu and Maharashtra, India	Case control 18 months	FSW N = 9111	Comparison between (Avahan and non-Avahan) intervention exposure: either active or passive. Distinction between metropolitan and ‘rest of state’ areas. Propensity score matching including matched controls unexposed to intervention	Trainings and meetings of FSWs, formation of self-help groups. Facilitating formation of CBOs. Fostering of capacity building and power negotiation.	NA	Increases in consistent condom use with all clients associated with level of participation: Attending a training session: Rest of Tamil Nadu (*p* < 0.001) and rest of Maharashtra (*p* = 0.008). Belonging to a self-help group: Rest of Tamil Nadu (*p* < 0.001) and Mumbai (*p* < 0.001). Belonging to a FSW collective: Mumbai (*p* = 0.013). No significant effects for Chennai.	Except in Mumbai, joining meeting or training associated with collective efficacy and community support. Belonging to a self-help group associated with collective efficacy in the rest of Tamil Nadu and Mumbai. Belonging to a self-help group associated with community support in the rest of Tamil Nadu and rest of Maharashtra
Ng et al. [60]	Avahan Six Indian states: Nagaland, Manipur, Tamil Nadu, Maharashtra, Karnataka and Andhra Pradesh.	Case control 2003–2008	High-risk groups (FSW, their clients and partners, MSM, IDU, and truck drivers). N = 626,232(?)	Comparison between intensity of Avahan by district (amount of grant per HIV population) using National Family Health Survey	Peer outreach for safe –sex counselling. Clinical services. Distribution of free condoms. Needle and syringe exchange. Community mobilisation and advocacy activities.	Greater programme intensity associated with lower likelihood of HIV prevalence in Andhra Pradesh (effect size −0.0026, 95 % CI −0.0044 to −0.0009, *p* = 0.004), Karnataka (effect size −0.0026, 95 % CI −0.0042 to −0.0008, *p* = 0.004) and Maharashtra (effect size −0.0022, 95 % CI −0.0039 to −0.0005, *p* = 0.008). Non-significant association in the other 3 states.	NA	NA
Ramesh et al. [61]	Avahan Five districts within Karnataka state: Mysore, Belgaum, Shimoga, Bellary and Bangalore Urban	Cohort Baseline: 7–19 months after project initiation; follow-up: 28–37 months later.	FSW N = 2312	Baseline and follow-up	Participatory mapping and enumeration exercises. Peer-mediated outreach and behaviour change communication. Dedicated sexual health services with STI syndromic management. Advocacy with stakeholders. Creation of drop-in centres (safe spaces for dialogue and services). Community mobilisation and capacity building.	Reductions prevalence of HIV (aOR 0.81, 95 % CI 0.67–0.99, *p* = 0.04), high-titre syphilis (aOR 0.53, 95 % CI 0.37–0.77, *p* = 0.001) and chlamydia and/or gonorrhoea (aOR 0.72, 95 % CI 0.54–0.94, *p* = 0.02). No differences in syphilis (aOR 0.77, 95 % CI 0.57–1.04, *p* = 0.09).	Increases in condom use at last sex with repeat clients (aOR 1.98, 95 % CI 1.58–2.48, *p* < 0.001). No differences in condom use at last sex with occasional clients (aOR 1.22, 95 % CI 0.89–1.66, *p* = 0.2) and regular partners (aOR 1.07, 95 % CI 0.76–1.51, *p* = 0.7).	NA
Reza-Paul et al. [62]	Avahan Mysore district only	Cohort Baseline: 6 months after project initiation, follow-up 2.5 years later	FSW N = 429	Baseline and follow-up	Participatory mapping and enumeration exercise. Community mobilisation and peer-mediated outreach. Increased access to sexual health services, expansion of condom availability in non-traditional outlets. Creating an enabling environment to support the programme.	No difference in HIV prevalence (aOR 0.91, 95 % CI 0.66–1.23) Increases in HSV-2 prevalence (64.4–79.0 %, aOR 2.15, 95 % CI 1.46–3.18, *p* < 0.001) Decreases in STI prevalence: syphilis (aOR 0.38, 95 % CI 0.25–0.59, *p* < 0.001); trichomonas (aOR 0.28, 95 % CI 0.18–0.41, *p* < 0.001); chlamydial infection (aOR 0.52, 95 % CI 0.27–0.96, *p* = 0.04) and gonorrhoea (aOR 0.42, 95 % CI 0.17–1.01, *p* = 0.03).	Increases in condom use: at last sex with occasional clients (aOR 4.30, 95 % CI 2.80–6.62, *p* < 0.001); with repeat clients (aOR 1.76, 95 % CI 1.22–2.55, *p* = 0.003); with regular partners (aOR 5.49, 95 % CI 2.91–10.37, *p* < 0.001). “Zero unprotected sex acts in past months” increased (aOR 5.45, 95 % CI 3.82–7.79, *p* < 0.001)	NA
Saggurti et al. [57]	Avahan Andhra Pradesh, India	Case control 2003–2010	FSW and men who have sex with men and transgenders (HR-MSM) FSW = 3,557 HR-MSM = 2,399	Comparison between low and high degree of 4 CM indicators: collective efficacy (both groups), collective agency (only FSW), collective action (only FSW), and participation in public events (only HR-MSM)	“[T]he implementation of community mobilization varied, with the differences mainly in group structures and focus on the local priorities and needs of communities”.	NA	FSW: High collective efficacy associated with greater likelihood of consistent condom use (CCU) with occasional (aOR 1.3, 95 % CI 1.1–1.7) and regular (aOR 1.4, 95 % CI 1.1–1.9) clients, and with STI treatment at government health facilities in past year (aOR 3.3, 95 % CI 2.1–5.1) High collective agency associated with greater likelihood of STI treatment only (aOR 1.6, 95 % CI 1.1–2.2) Collective action associated with greater likelihood of CCU with occasional (aOR 1.3, 95 % CI 1.1–1.8) and regular (aOR 1.5, 95 % CI 1.1–2.0) clients, and with a lower likelihood of STI treatment (aOR 0.5, 95 % CI 0.3–0.8). HR-MSM: participation in public event associated with greater likelihood of CCU with paid (aOR 3.3, 95 % CI 2.1–5.2) and paying (aOR 2.7, 95 % CI 2.0–3.6) partners. Higher collective efficacy associated with greater likelihood of CCU with paying partners. (aOR 1.9, 95 % CI 1.5–2.3) only.	NA
Blanchard et al. [58]	Avahan Districts in Karnataka (Belgaum, Gulbarga, Gadag, Dharwad) and Maharashtra (Solapur), India	Case control	FSW N = 1,750	Comparison between high intensity intervention (Belgaum, Gulbarga and Gadag) and low intensity intervention (Dharwad and Solapur) districts. Analysis by dimensions of empowerment (within, with and over) Only power with (collective identity and solidarity) included here	CM through an “integrated empowerment framework”: sex work organisation, program and structural interventions, sociodemographic characteristics impacting upon empowerment dimensions, which in turn result into the power to address a disempowering social context (power imbalances, social exclusion and vulnerability). Sensitisation of stakeholders in and beyond the community.	NA	In both high and low intensity districts, power with was associated with likelihood of condom use at last sex with regular client (aOR 2.56, *p* < 0.001; aOR 1.65, *p* < 0.01) and frequency of condom use with regular clients (aOR 2.27, *p* < 0.001; aOR 1.80, *p* < 0.001), with no significant differences in condom use at last sex with regular partner, frequency of condom use with regular partner and number of visits to health clinic for health problems.	Power with was associated with lower odds of violence or abuse by more powerful groups in high intensity districts (aOR 1.34, *p* < 0.05), but not in low intensity ones.
Parimi et al. [64]	India HIV/AIDS Alliance programme Andhra Pradesh, India	Case control 2004–2010	FSW in 5 districts of Andhra Pradesh N = 1,986; n = 1116 from project areas where STI services were in partnership with government	870 FSW (43.8 % of sample) from project areas where STI services were delivered by other (agency-implemented or in partnership with private providers) STI service delivery models	Promotion of governmental STI services utilisation. Syndromic management of STIs among government facilities. Sensitisation meetings with outreach staff and community members. Community-based group promotion of health care facilities use among FSW. Awareness camps focused on risk perception: games, street plays, puppet shows and magnet theatre shows.	NA	Regardless of project area, FSW who reported high collectivisation (collective-efficacy, -agency and -action) were more likely to access STI treatment from government health facilities in the previous year than their low collectivisation counterparts (78.1 *vs* 63.2 %, aOR 2.1, 95 % CI 1.6–2.8)	NA

*aOR* adjusted odds ratio, *aIRR* adjusted incidence rate ratio, *aPR* adjusted prevalence ratio, *aRR* adjusted risk ration, *BL* baseline, *CI* confidence intervals, *EL* endline, *IRR* incidence rate ratio, *MN* McNemar value, *NA* non-applicable (not measured), *nI* intervention sample, *nC* control sample, *POR* prevalence odds ratio, *RRi*: Relative risk of infection, *SD* standard deviations

^a^Reported according to classification in the Quality Assessment Tool for Quantitative Studies Dictionary

^b^When preparatory work involved the community, this has been counted as part of the intervention components

^c^For the MEMA kwa Vijana trial, sample numbers are reported for endline, given that the article by Doyle and colleagues (2010) is a long-term follow-up

Outcomes related to knowledge, attitudes, individual level perceptions (e.g. attitude towards condoms, self-efficacy or individual-level ‘power-within’) were not included, given that knowledge, individual skills and attitudes are not a sound reflection of behaviour [42–44]. Similarly, *reported* STI were omitted on the basis that an actual measure (incidence/prevalence), rather than a proxy, was more valid. We included results for different types of outcomes if reported in more than one article belonging to the same study. When similar outcomes for the same study were reported by more than one article, we included the results using a larger sample, given that this frequently aggregated the smaller samples reported elsewhere.

Given the heterogeneity of studies in terms of study design, intervention participants and outcomes measurement, a meta-analysis would have been unsuitable for this review. Consequently, the narrative analysis presented below addresses our review questions and, for the second one, relies on both the *direction* of intervention effects and associations and their reported *significance*. Furthermore, although studies’ risk of bias did not determine inclusion, we assessed this risk and methodological soundness by using Thomas’s Quality Assessment tool for Quantitative Studies [45]. This instrument is recommended for systematic reviews of health interventions [46] and, while suitable for our review because it evaluates a range of quantitative designs [47], was adapted to accommodate the complexity of studies included (Table 2).

**Table 2:** Quality assessment

STUDY	Assigned design^a^	Selection bias	Study design	Confounders	Blinding	Data collection method	Withdrawals and dropouts	Global rating
Doyle et al. [50]	RCT	Strong	Strong	Strong	Moderate	Strong	Moderate	Strong
Kamali et al. [54]	RCT	Strong	Strong	Strong	Moderate	Strong	Moderate	Strong
Cowan et al. [49]	RCT	Moderate	Strong	Strong	Moderate	Strong	Weak	Moderate
Gregson et al. [52]	RCT	Moderate	Strong	Strong	Moderate	Strong	Weak	Moderate
Jewkes et al. [51]	RCT	Weak	Strong	Strong	Moderate	Strong	Moderate	Moderate
Pronyk et al. [53]	RCT	Moderate	Strong	Strong	Moderate	Strong	Weak	Moderate
Sweat et al. [15]	RCT	Moderate	Strong	Weak	Weak	Strong	Moderate	Weak
Kerrigan et al. [14]	Cohort analytic	Moderate	Moderate	Strong	Moderate	Strong	Moderate	Strong
Gao and Wang [72]	Cohort analytic	Moderate	Moderate	Strong	Moderate	Weak	Strong	Moderate
Swendeman et al. [65]	Cohort analytic	Strong	Moderate	Strong	Moderate	Weak	Moderate	Moderate
Erausquin et al. [56]	Cohort analytic	Moderate	Moderate	Moderate	Moderate	Weak	Strong	Moderate
Lippman et al. [67]	Cohort analytic	Moderate	Moderate	Strong	Moderate	Strong	Weak	Moderate
Gutierrez et al. [63]	Cohort analytic	Weak	Moderate	Strong	Weak	Strong	Weak	Weak
Gutiérrez et al. [69]	Cohort analytic	Weak	Moderate	Strong	Weak	Strong	Weak	Weak
Kerrigan et al. [70]	Cohort	Moderate	Moderate	Strong	Moderate	Weak	Strong	Moderate
Williams et al. [71]	Cohort	Weak	Moderate	Moderate	Moderate	Strong	Moderate	Moderate
Schensul et al. [39]	Cohort	Moderate	Moderate	Moderate	Moderate	Weak	Strong	Moderate
Benzaken et al. [68]	Cohort	Weak	Moderate	Moderate	Moderate	Weak	Weak	Weak
Ng et al.^b^ [60]	Case control	Moderate	Moderate	Strong	Moderate	Strong	Moderate	Strong
Parimi et al. [64]	Case control	Moderate	Moderate	Strong	Moderate	Weak	Moderate	Moderate

The following adaptations were performed to the instrument’s grading when assessing the studies: (a) Validity and reliability (measured under data collection method) were assigned as ‘strong’ for all studies using biomarkers. For studies relying on behavioural outcomes, explicit indication of the instrument’s validity was sought and reliability coefficients were required. (b) Baseline differences (assessed under confounders) were computed as ‘moderate’ for all cohort studies (one group pre + post (before and after)), given that they act as their own comparison group. (c) For studies that did not involve the same participants at baseline than at follow up, completion rate was computed by calculating the proportion of participants in the follow up in relation to those who participated at baseline. (d) For studies implementing the intervention in one community group but measuring effects at the population level, representativeness (under selection bias) was marked as ‘somewhat likely’

^a^Given the heterogeneity of individually reported designs, they were classified according to the instruments’ typology. Assigned design is also reported in the analytic table

^b^Data from Ng and colleagues’ (2011) article were used for the assessment of this study because they encompass the Indian states considered in the other articles reviewed

## Results

### The Studies

Of the corpus of twenty studies, seven were RCT: project Accept [15, 48], the Regai Dzive Shiri intervention [49], the MEMA kwa Vijana trial [50], the Stepping Stones intervention [51], the Manicaland Project [52], the IMAGE project [53], and a trial in the Masaka district, Uganda [54]. Project Accept was carried out simultaneously in Sub-Saharan Africa and South and South East Asia, while the other six trials were implemented in Sub-Saharan Africa.

The remaining twelve studies used various observational designs. In India, South and South East Asia, the RISHTA project [39], the Parivartan project [55, 56], the Avahan project [57–62], the Frontiers Prevention Project (India) [63], a programme by the India HIV/AIDS Alliance [64], as well as a Sonagachi-replication [65, 66] were implemented; in Latin America and the Caribbean, the Encontros project [67], the Princesinha project [68], the Frontiers Prevention Project (Ecuador) [69], a Sonagachi-inspired intervention [70], and a comparison between CM and CM plus policy changes [14] were carried out. Finally, the Carletonville project [71] was undertaken in Sub-Saharan Africa and a participatory intervention was implemented in Chengdu, China [72].

The boundaries of ‘community’ were conceptualised in three main ways by the selected studies. First, in contexts of concentrated HIV epidemics, interventions targeted groups most at risk: ten studies focused on sex workers [14, 56, 59, 63–65, 67–70], four on men who have sex with men (MSM) [57, 63, 69, 72], and one study [39] focused on local heterosexual men whose high levels of alcohol consumption were found to be putting their sexual health at risk [73]. These communities were thus assumed to share a social identity, location and concrete practices (e.g. work and leisure). Second, mainly in contexts of generalised epidemics, youth were targeted by four studies [15, 49–51], treating them also as communities in terms of identity and sexual risks, within geographically-bound communities. Four further studies [52–54, 71] conducted in the generalised epidemic of Sub-Saharan Africa were concerned with mobilising geographically-bound communities by targeting adults or a number of groups (e.g. women who applied for a loan; miners, sex workers and adults simultaneously) within the community. Outcomes were evaluated at the level of participant communities and their comparators, except for four projects (Accept, Avahan, IMAGE and Regai Dzive Shiri), which evaluated the effects of the intervention at the wider community- or population-level.

### Do CM Interventions Work? It Depends on for Whom

Since the pattern of findings differs by population, we have divided our presentation of the findings into two sections, reporting first the findings for sex workers and other most at risk groups, and then findings for youth and general communities.

#### Sex Workers and Other Most at Risk Groups

The first group are mainly sex workers and, to a lesser extent, other most at risk groups such as men who have sex with men (MSM), who have been targeted in contexts of concentrated HIV epidemics. Inconsistent results were reported in the Avahan programme, in which for population effects at state level, “greater intensity” of the intervention was significantly associated with lower HIV prevalence in 3 Indian states, but with very small effect sizes (−0.0026 to −0.0022). The association between CMI and HIV prevalence was non-significant in 3 other states [60–62], although authors acknowledge that the prevalence in chronic diseases such as HIV could require long periods to be apparent. The Frontiers Prevention Project in Ecuador found no significant effects of the intervention on HIV seroprevalence among FSW and MSM [69].

With regards to other STI, a sub-study of the Avahan programme in Karnataka reported that chlamydia and/or gonorrhoea prevalence, and high-titre syphilis, were significantly reduced, while this reduction was non-significant in relation to syphilis among female sex workers (FSW) [61]. Encouragingly, the Frontiers Prevention Project in Andhra Pradesh was associated with lower likelihood of syphilis and HSV-2 among both FSW and MSM [63]. A similar project in Ecuador rendered a significant impact in the reduction of likelihood of syphilis seroprevalence among MSM, while having a borderline effect of lower HSV-2 seroprevalence among FSW in the programme [69]; non-significant programme effects were observed on HSV-2 among MSM and syphilis among FSW [69]. Active participation in the Encontros study was related to a non-significantly lower probability of incident chlamydia and/or gonorrhoea [67], while the Carletonville project rendered a non-significant increase of syphilis, gonorrhoea and chlamydia among participant sex workers [71]. Furthermore, the study in the Dominican Republic [14] found CMI to reduce prevalence of one or more STI (gonorrhoea, trichomoniasis, or chlamydia) among FSW. A further analysis shows that this effect was statistically significant when CM was combined with implementing and enforcing a government policy supporting consistent (100 %) condom use [14]. On the whole, the evidence points to CMI tending to impact on the reduction of STI *among sex workers*, with this effect being more likely to be significant when CMI is combined with policy interventions [14].

Regarding behavioural outcomes, among sex workers, condom use is the behaviour most addressed, and with the strongest evidence, with various degrees of effect depending on whether sexual encounters are with paying clients, casual or stable partners. Exposure to interventions was found to be significantly associated with increased likelihood of condom use [66], consistent condom use with clients [56], with new clients [14 in Santo Domingo], consistently or during last encounter with regular clients or partners, [14 in Puerto Plata, 61, 63, 67], and for oral sex with clients, in “all situations” and during last week [68]. Other studies have found non-significant or marginal increases related to the intervention, including ever using a condom [71], condom use with last client [63, 70], with all clients [70], with occasional or new clients [14 in Puerto Plata, 61, 67], with non-paying [67] and regular partners [14 in Santo Domingo, 61, 69], as well as for anal and vaginal sex with clients [68]. Marginal decreases in consistent condom use with all partners [70] and casual partners [71] were reported in Rio de Janeiro, Brazil and South Africa.

Of note are two behavioural outcomes reported in the interventions with FSW. In the Dominican Republic [14], authors recorded the *observed* FSW’s verbal rejection of unsafe commercial sex, which increased from pre- to post-intervention in both sites, being significant only for the community where both CM and policy enforcement were implemented. Similarly, in Andhra Pradesh [64], FSW who reported high collectivisation were considerably more likely to procure STI treatment from government health facilities than those who reported low collectivisation, as were those who reported high collective efficacy and collective agency [57]. Collective action, in contrast, was associated with a lower likelihood of STI treatment-seeking [57].

CMI targeting MSM were found to be related to a significant increase in reported condom use with casual and regular sexual partners for vaginal sex, anal sex and oral sex [72] and to the likelihood of condom use with last female partner [63] and last male sexual partner [69]. An association in the same direction was non-significant for condom use with last female partner [69] and last male sexual partner [63]. In one subsample of MSM and transgender people in the Avahan project [57] it was found that participation in a public event was significantly associated with higher likelihood of consistent condom use among paying and non-paying partners, with the same positive trend for collective efficacy, although significant only with paying partners. In the RISHTA intervention [39], which engaged local heterosexual males whose high levels of alcohol consumption were found to put their sexual health at risk, it was reported that changes in extramarital sex were significantly associated to change in alcohol use, so that significant decreases in extramarital sex were observed among men who were drinkers at baseline but non-drinkers at endline.

Social outcomes tended to be positive mainly in terms of community participation and collective identity, with inconsistency in the way social outcomes were measured. Two Sonagachi-inspired programmes found significantly positive changes in social participation [67, 70], but not in perceived social cohesion [67, 70] after intervention in Corumbá [67] and Rio de Janeiro [70], Brazil. Similarly, another Sonagachi adaptation found significant increases in social support through organising and solidarity, but not in political participation in West Bengal, India [65]. An evaluation of the Avahan project, in turn, found that joining a meeting, belonging to a help group and being member of a sex worker collective were significantly associated with higher perceived collective efficacy and higher perceived collective support in non-metropolitan Tamil Nadu, with varying positive effects in the other three settings of Tamil Nadu and Maharashtra, in India [59]. A different evaluation report of Avahan also found that collective identity and solidarity were significantly associated with lower odds of violence or abuse by more powerful groups in high-intensity intervention districts, but not in low-intensity ones [58]. In Andhra Pradesh, the study on project Parivartan found a positive relationship between collective identity, collective efficacy and collective agency and programme exposure, which becomes stronger as the level of exposure rises [55].

In sum, there is reasonable evidence for the effectiveness of CMI in reducing STI and increasing condom use among sex workers. Among MSM, there is some evidence that CMI results in increased condom use. Findings regarding social outcomes remain uneven, depending on the social outcome measured. The evidence of CMI effects on HIV prevalence remains limited to projects Avahan and Frontiers Prevention (Ecuador), which provided inconclusive results. It is difficult to draw broad conclusions about which programmatic elements or conditions are most effective (e.g. targeting casual vs. regular partners; working in urban vs. rural areas) because each intervention employed a different design, measured different outcomes, and was conducted in a unique setting.

#### Youth and General Community

Studies targeting either youth or the general community reported mainly non-significant intervention effects in terms of HIV incidence [51–54] or prevalence at the community level [49, 50]. Project Accept intervention reported lower HIV incidence in intervention than in control communities with borderline statistical significance (*p* = 0.08) [48]. Similarly, regarding other STI, mainly non-significant effects have been reported for HSV-2 prevalence [49, 50] and incidence [54 for one arm] as well as prevalence of chlamydia, syphilis and gonorrhoea [50, 54 for one arm], while the Carletonville study reported significant *increases* of chlamydia among miners, men and women, of syphilis among miners and women, and of gonorrhoea among miners [71]. The Masaka trial in Uganda, in turn, found dissimilar results depending on intervention arm: incidence of active syphilis and prevalence of gonorrhoea were significantly lower in one of the intervention arms than in the control group, while HSV-2 incidence was lower in the other intervention arm than in the control group [54]. Only the Stepping Stones programme was associated with a significantly lower HSV-2 incidence in comparison with controls [51]. Hence, there is little evidence that CMI succeed in reducing numbers of HIV and/or STI cases among youth and general communities, with some success limited to the Stepping Stones programme and project Accept.

In terms of behavioural markers, CMI were found to be significantly positively associated with the likelihood of condom use with casual partners [50 in females, 54 in one arm; 71 in miners, men and women] and ever using a condom [71 in miners and women]. However, for a number of interventions no significant changes in either direction were reported on condom use at last sex [49–51], reported ever condom use [54, 71 in men] and condom use with regular [52] and non-spousal [53] partners. For the Manicaland study [52], reported condom use with casual partners was significantly more common in control than in intervention communities. Behaviours other than condom use were also addressed. In the Accept study, the proportion of people taking their first HIV test was significantly larger in community based voluntary counselling and testing communities than in standard care areas [15]. However, the IMAGE project found non-significant differences in having had an HIV test in intervention groups relative to controls [53]. Similarly, in terms of health service use, the Regai Dzive Shiri project had no effect on clinic attendance [49] and the Manicaland project found no intervention-related differences in treatment-seeking within 3 days of STI symptoms [52]. In light of these results, CMI appears to have some effect among youth and targeted communities on condom use with casual partners and promising effects in the uptake of voluntary testing, although evidence for the latter is limited to one study.

Among programmes targeting youth and the general population, evidence of effects on social outcomes is limited to the IMAGE and the Stepping Stones projects. In the former, targeted women reported a significant reduction in intimate partner violence and were more likely to report higher levels of participation in social groups and collective action, than their comparison counterparts, with no significant differences regarding the perception of solidarity and that the community would work together to achieve common aims [53]. In the Stepping Stones programme the proportion of male participants who reported enactment of intimate partner violence was lower than among controls, with this trend maintained across the intervention’s lifetime (*p* = 0.099, *p* = 0.054 at 12 and 24 months, respectively), although there was no evidence of this difference among women [51]. Therefore, while these results are promising, there still remain inconsistencies regarding their diffusion into biomedical and behavioural indicators.

In sum, for studies involving youth, targeted groups within communities and geographically-bound communities, no significant results were found for reductions of HIV incidence or prevalence, while marginal impact on the reduction of other STI was identified, mirroring the results of previous reviews [33]. The evidence suggests that while these programmes impact on reported condom use with casual partners, this improvement may not translate into significant changes in biomedical markers. The results obtained for social outcomes are fairly positive but limited to two studies, and hence their relationship with behavioural and biomedical indicators remains to be clarified.

## Discussion

The present review has gathered evidence of the effectiveness of interventions with a CM component on biomedical, behavioural and social outcomes. We present our discussion in two sections. The first assesses what can be learnt from our review to inform contemporary CM programming. Given that the findings are generally inconclusive, the second section critically reflects on the literature, to explore reasons for the inconclusiveness of the evidence.

### The Systematic Review: What has been Found?

Overall, this systematic review has produced a somewhat inconclusive set of findings. Among sex workers and groups most at risk, the evidence bears some degree of consistency, indicating an overall tendency of positive impact, with more consistent and stronger results for behavioural and social outcomes than for biomedical ones. Among youth and general communities, the evidence of the effects of CMI remains inconclusive. Overall, it is not possible at this point in time to come to a general conclusion as to whether CMI are effective or not, though there is suggestive evidence for sex worker groups. Our review suggests, nonetheless, two more nuanced lessons that may be drawn.

The first is that CMI appear to be more successful with groups who have a meaningful collective identity rather than with more generalised populations. One of the main characteristics of interventions engaging sex workers and groups most at risk is that they capitalise on these groups’ collective identity. CMI often work through their situation of vulnerability to foster mobilisation that is cohesive and fuelled by a need not only to attain HIV-related goals, but also to increase their material and symbolic power and status in the community. Indeed collective identity could arguably have been one of the reasons for success of organic forms of CM efforts such as Sonagachi [74].

One explanation for the different approach in these programmes is that young people and general communities do not evidently display the extreme and conspicuous disadvantages of sex workers and thus do not appear in need of tackling the social determinants of their problems. It is plausible that sex workers can mobilise against specific structural factors that marginalise them particularly (policies, structures and laws to deter sex work), which is not the case with general populations. Similarly, it is possible that mobilising a sub-set of a population is easier than mobilising entire communities. For the case of youth, hence, if they were discriminated against (as is the case of MSM in some contexts), this might foster a collective identity in the group, which would in turn facilitate tackling identifiable social determinants of health among this group.

Second, CMI seem more likely to generate favourable outcomes if accompanied by efforts for change at the structural level. For example, Kerrigan and colleagues’ study in the Dominican Republic provided evidence that CMI alone renders some positive outcomes, but when implemented alongside structural changes such as brothel policy of 100 % condom use its results were more effective [14]. Similarly, researchers of the MEMA kwa Vijana trial identified the low status of young people in the community as a barrier to attaining better results as well as females’ lower social status and financial reliance on males [75]. These factors, among other sociocultural issues identified by researchers, point to the need to work not only with the ‘target group’ but also with other community groups, in order to tackle structural barriers to CMI effectiveness.

### Critique: Why are the Findings Inconclusive?

We suggest that the evidence is inconclusive not because CMI are ineffective, but instead due to problems with operationalization, evaluation and review methodologies. In other words, the full potential of CMI has rarely been evaluated. In what follows, we discuss problems in the literature, at the level of operationalization of CM, the attention to socio-political context, and the nature of review methodologies. While these problems afflict some parts of the literature, various authors have actively sought to address the problems appropriately. Thus, following each problem we also discuss ways of pre-empting or mitigating such problems.

First, inconclusive results may be related to the *operationalization of CM.* For complex interventions and trials in general there are a number of programme design issues that impinge on intervention impact, such as programme length, follow-up timespan, intervention exposure and adherence, as well as “underpowered” designs, as has been previously pointed out [33, 50, 60, 76]. In this review, we have identified three flaws in the operationalization which we discuss in turn: (i) understandings of CM remain underdeveloped, and often tokenistic; (ii) implementations of CMI are often characterised by inflexibility; and (iii) the evaluation of CMI tends to inadequately account for social impact.

The first point about operationalization concerns the degree to which CM interventions allow for genuine community ownership. In theory, the merit of CM lies in building sustainable community strengths and agency at the community level [77, 78]. In practice, however, the concept is often used to refer to *static and tokenistic activities* in which researchers gather “the community” and establish contact with relevant stakeholders. Despite our efforts to employ appropriate inclusion criteria, limited versions of CM were employed in several of our reviewed studies. This was particularly notable in interventions with youth and general communities. Articles describing the nature of CM in the reviewed studies included statements referring to CM as “community sensitisation…to inform the community about the study” and to obtain authorisation [79], activities “to reduce opposition” to the intervention programme [75], “the process of gaining community support for the study” [80], and undertakings to ascertain leaders’ “views and seek their support in encouraging community participation” [81]. In these instances, interventions draw on local knowledge and input to execute programmes planned by outsiders [82]. In such cases, at best, communities are “mobilised” first, to *gain access* to their networks and thus enable research execution and second, to participate in *programme delivery* [82]. They may not in fact be building and capitalising on the community connections that comprise the main rationale for CM.

Examples of projects that managed to operationalize CM in a way that fostered supportive community relations come from those targeting sex workers and groups most at risk. Overall, these interventions included activities that triggered active community engagement through de-stigmatising public events, fostering of within-community cohesion and alliances with external stakeholders. The Princesinha project in Brazil [68], for example, engaged sex workers as leaders of project activities and data collectors. Public exposure was raised through celebrating the eldest sex worker, carnival participation through a “prejudice-free” samba group carrying prevention posters [68]. In a similar attempt in Brazil, the Encontros project [83] included activities that allowed within-community dialogue around sex work, discrimination, human rights and HIV/STI prevention. They also organised “hot-pink” parties, cultural performances by sex workers at the city’s cultural centre, along with external partnerships with the community at large [83]. What these and other interventions [e.g. 72] have in common is the thoughtful implementation of activities that are inclusive of community members and build cohesive relationships among them, while fostering their self-presentation as an assertive ‘community’ in negotiations with stakeholders in the public sphere.

Stemming from this understanding of CM and in line with requirements of standardisation of intervention components in evaluation research, the second problem at the level of operationalization is *inflexibility* in the way the majority of the programmes included in our review responded to the needs of communities. A premise of CM is that interventions must be appropriate, and thus adapted to specific local contexts based on community ownership and leadership [2, 18]. However, when studies reported changes in the planned implementation and evaluation, this was presented as a remedial measure taken *by researchers*, which limits meaningful engagement of the target community and therefore ownership of the project’s objectives. For instance, the Manicaland project did not implement the income-generating intervention component originally planned because of country-wide economic decline during the trial [52]. While Stepping Stones programmers acknowledged that “development of interventions is an iterative process, and interventions are generally strengthened by being more extensively tested and adapted” [51], adaptations to the original intervention occurred *before* this trial was implemented and to fulfil research needs rather than community demands [51].

Among the studies included in this review, two interventions made explicit adaptations while implementing CM. Project Accept [84] made an explicit programmatic point of allowing “site-specific adaptations” to accommodate “site-specific sociocultural differences” in its varying settings. Researchers developed a thoughtful way of balancing consistency and flexibility while maintaining a “minimum level of comparability” [85]. Strategies used to enable consistency of themes across adaptations included engaging field staff in producing the adaptations, ensuring community acceptance, and using steering committee, ethical review boards and intervention subcommittees to approve and implement adaptations [85]. The Avahan intervention also documented the changes applied according to community demands during its implementations [86]. The remaining challenge, of course, is that such complexity, changes, and relative lack of control are at odds with the requirements of rigorous and internally-valid designs such as RCT.

The third problematic point regarding the current operationalization of CM concerns *methodological issues in the measurement of impact*, particularly *social impact*. The choice of impacts to measure, and of measurement tools, is often weak, particularly for social outcomes. There might be benefits gained by CMI participants that are not necessarily part of the programmes’ evaluated outcomes (e.g. health service use) or that are intangible (e.g. increased participation in groups outside the ‘target’ community). Among interventions with sex workers, some programmes [55, 59] limit the appraisal of social outcomes to one question per dimension (e.g. collective efficacy), restricting the power of such measurements. This indicates the need both to improve quantitative instruments, and to triangulate evidence from more open-ended data collection methods, to maximise learning from an intervention.

For example, some of the studies included in this review have used process evaluation to explain their quantitative effects [75, 87] to document the challenges of implementing RCT among deprived, rural groupings [81], and to report the most successful CM approaches to engage communities [88]. Process evaluation represents a viable option to gauge the social transformations triggered by CMI because it documents the context of the ‘black box’ that often seems to be present in ‘input–output’ models. In addition, it documents ‘achievements’ that are part of the intervention per se. This is the case because in many contexts the sheer implementation of the programme might be in fact contesting the status quo of its target population, which was the case of a number of studies in this review [67, 68], but that is often missed in quantitative evaluations such as those included here.

Second, many of the interventions *failed to engage with the broader social and political context* and power relations that structure health in very disadvantaged communities [2]. Contemporary understandings of CM emphasise that communities alone rarely have the power to make the social changes needed to sustain healthy behaviour, and hence, that alongside CM, efforts to engage powerful stakeholders and to move towards structural changes are also required [18, 27, 89]. In contrast to these understandings, in programmes involving youth and general communities, there was evidence of limited efforts to engage the broader community. Where efforts were made to engage groups beyond the target group, this often had the limited aim of enabling the diffusion of health-related knowledge, to parents or other groups [53, 79] rather than engaging them in transformative change.

Among our reviewed studies, it was notable that interventions with sex workers often took greater account of the socio-political context. In such studies, having a support network, altering community relationships and fostering collective action have the potential to bring much wider benefits and thus be valued in their own right, beyond their contribution specifically to HIV prevention. For instance, advocacy was conducted with the police, local government officials, community leaders, FSW’s partners and clients, and other gatekeepers [62, 66]. In this way, the ‘community’ that brings about the project is more inclusive than the interventions’ target community groups [23].

Third, reflecting on the very uneven nature of the findings, we suggest that the goal of *providing an over*-*arching statement of ‘the evidence’ for CM may itself be misguided* [90, 91]. Most obviously, we have noted that there is a different pattern of findings for sex workers and for youth and general communities. We have observed that in some studies, there appear to be impacts on condom use with some types of partners, but not others. The IMAGE study found impressive effects on intimate partner violence (and this is widely argued to be a likely contributor to HIV transmission), but no effects on HIV incidence, which by its nature is more difficult to assess. Based on earlier positive results from the Sonagachi Project [16, 92], replications were implemented in Brazil [67, 70] and India [65, 66] but to less positive effect. Furthermore, the Avahan intervention has disaggregated CM components and their impact on a host of measures of condom use in a variety of settings, finding some significant relationships at a fine-grained level, but not much consistency across results [57–59, 61, 62].

Such inconsistent findings make it appear unrealistic to expect a singular statement about whether CM ‘works’ or not. More nuanced statements, about the conditions under which CM is more likely to work might have greater potential (e.g. our review suggests that CM may be more likely to succeed if it is implemented in tandem with policy changes). However, it seems unlikely that a definitive set of decision rules to determine when CM should be attempted could be achieved. CM is, by its very nature, contextual and evolving. CM mobilises contextually-specific local networks, in locally-appropriate ways, and allows communities power to create and alter objectives. Thus, CM is not simply an intervention that is equivalent across sites, but takes different forms in different sites. Although the ‘evidence-based policy and practice’ paradigm prioritises controlled trials and systematic reviews of these, it may be that multi-faceted and context-specific CMI are more challenging to quantify, compare and appraise.

### Implications

The above critical discussion has implications for future implementation and evaluation of CM. The first is the need for operationalization of CM informed by a committed understanding of social change. We are concerned that the evaluated CMI may not in fact be a good test of the effectiveness of CM, because the interventions do not always heed the transformative objectives behind CM, but treat it simply as an instrumental add-on to increase uptake, being inflexible to the contextual needs of the community participating in the intervention, and using simplistic measures of social outcomes. Part of the issue may be that the improvisational and responsive nature of genuine CM is not compatible with the methodological requirements of controlling variables and standardising intervention components. Another possibility is that the biomedical professionals who often lead such interventions are not equipped with the skills to facilitate an open-ended and complex social process of mobilisation [2, 93]. We propose that a clear understanding of CM, informed by a social scientific theory of change, and recognising the need for specific community development skills is needed. The more established our understanding of CM is, the less likely it will be that the concept is stripped-down and depoliticised when operationalized.

Second, our discussion of context and social groups points towards the need to work with communities to address the socio-political context and to build supportive partnerships with more powerful groups, rather than with community groups in isolation. An enabling policy environment (e.g. decriminalisation and de-stigmatisation of sex work and homosexuality, governmental policies for participatory community planning of interventions) is required for communities to address socio-political issues. The reviewed studies targeting sex workers illustrate that, when such an enabling environment is absent, advocacy may be needed as part of CMI in order to negotiate power relations.

Finally, our critique of the systematic review methodology suggests that judgements about the suitability of CM may need to be made on a more local basis, and informed by a wider set of evidence than that provided by systematic reviews and/or rigorous outcome evaluations. Contemporary work in the philosophy of science questions the desirability of conceptualising social interventions in terms of ‘replication’ across diverse contexts, arguing that “to draw causal inferences about a target population, which method is best depends case-by-case on what background knowledge we have” [94]. The implication here is that a systematic review of outcome evaluations is insufficient information on which to base the choice or design of a CM intervention. Such information needs to be combined with other sources, including a plausible theory of change and knowledge of the particular context into which the intervention is being introduced.

## Conclusion

Taking the evidence at face value (irrespective of our critiques of the form of this evidence), it seems too early to decide whether CM works or not, especially considering the heterogeneity of interventions. At present, at least two RCT which explicitly include CM as a component are being conducted and awaiting biomedical results [95, 96]. They may offer further evidence of the contribution of these quantifying approaches to the planning, implementation and evaluation of CM as currently conceptualised. However, taking our critiques seriously, we suggest that the very aspiration to provide a single statement of ‘the evidence’ for diverse, evolving, and multifaceted CMI in complex settings may be misguided.

## Electronic Supplementary Material

Below is the link to the electronic supplementary material.

Supplementary material 1 (DOCX 20 kb)
