# Supplementary material for: The impact of Community Mobilisation on HIV Prevention in Middle and Low Income Countries: A Systematic Review and Critique
Source: AIDS Behav. 2014 Mar 23;18(11):2110–34. doi: 10.1007/s10461-014-0748-5 (PMC4196137; doi:10.1007/s10461-014-0748-5)
Supplement: Supplementary file 1 — Supplementary material 1 (DOCX 20 kb) [file 10461_2014_748_MOESM1_ESM.docx]

**Rationale behind search strings:** Searches were performed individually for each of the seven key terms (i.e. “community mobili*”, “community particip*”, “community led”, “community based”, “community activit*”, “community development”, “capacity building”) to systematise the number of records rendered by each term. This would give us an indication of the extent to which community mobilisation is explicitly called as such rather than by other terms.

**Note:** The search was originally performed in 2012 and updated in November 2013, after first submission of the article for review. For the sake of brevity, complete strings are presented as a single search. Registers were kept of all individual strings and records computed for each search round.

**SCOPUS**

TITLE-ABS-KEY("community mobili*" AND hiv OR aids) AND PUBYEAR > 2002

## TITLE-ABS-KEY("community particip*" AND hiv OR aids) AND PUBYEAR > 2002

## TITLE-ABS-KEY("community led" AND hiv OR aids) AND PUBYEAR > 2002

(TITLE-ABS-KEY("community based") AND TITLE-ABS-KEY(intervention) AND TITLE-ABS-KEY(hiv OR aids)) AND PUBYEAR > 2002

(TITLE-ABS-KEY("community activit*") AND TITLE-ABS-KEY(intervention) AND TITLE-ABS-KEY(hiv OR aids)) AND PUBYEAR > 2002

(TITLE-ABS-KEY("community development") AND TITLE-ABS-KEY(intervention) AND TITLE-ABS-KEY(hiv OR aids)) AND PUBYEAR > 2002

TITLE-ABS-KEY("capacity building") AND TITLE-ABS-KEY(hiv OR aids) AND TITLE-ABS-KEY(community) AND TITLE-ABS-KEY(intervention) AND PUBYEAR > 2002

**PubMed**

((community mobili*[Title/Abstract]) AND hiv[Title/Abstract]) AND ("2003/01/01"[Date - Publication] : "3000"[Date - Publication])

((community mobili*[Title/Abstract]) AND aids[Title/Abstract]) AND ("2003/01/01"[Date - Publication] : "3000"[Date - Publication])

## ((community particip*[Title/Abstract]) AND aids[Title/Abstract]) AND ("2003/01/01"[Date - Publication] : "3000"[Date - Publication])

## ((community particip*[Title/Abstract]) AND hiv[Title/Abstract]) AND ("2003/01/01"[Date - Publication] : "3000"[Date - Publication])

((community led[Title/Abstract]) AND hiv[Title/Abstract]) AND ("2003/01/01"[Date - Publication] : "3000"[Date - Publication])

((community led[Title/Abstract]) AND aids[Title/Abstract]) AND ("2003/01/01"[Date - Publication] : "3000"[Date - Publication])

(((community based[Title/Abstract]) AND intervention[Title/Abstract]) AND (hiv OR aids[Title/Abstract])) AND ("2003/01/01"[Date - Publication] : "3000"[Date - Publication])

(((community activit*[Title/Abstract]) AND intervention[Title/Abstract]) AND (hiv OR aids[Title/Abstract])) AND ("2003/01/01"[Date - Publication] : "3000"[Date - Publication])

(((community development[Title/Abstract]) AND intervention[Title/Abstract]) AND (hiv OR aids[Title/Abstract])) AND ("2003/01/01"[Date - Publication] : "3000"[Date - Publication])

((((capacity building[Title/Abstract]) AND intervention[Title/Abstract]) AND community[Title/Abstract]) AND (hiv OR aids[Title/Abstract])) AND ("2003/01/01"[Date - Publication] : "3000"[Date - Publication])

**CINAHL**

[AB "community mobili*" AND AB ( hiv OR aids )](http://web.ebscohost.com/ehost/breadbox/search?term=AB%20%22community%20mobili*%22%20AND%20AB%20(%20hiv%20OR%20aids%20)&sid=c1b2ba28-c406-4dc5-9c9f-26ecbd670bcf%40sessionmgr112&vid=5)

#### Limiters

Published Date from: 20030101-201231031

AB "community particip*" AND AB ( hiv OR aids )

Limiters

Published Date from: 20030101-20131031

[AB "community led" AND AB ( hiv OR aids )](http://web.ebscohost.com/ehost/breadbox/search?term=AB%20%22community%20led%22%20AND%20AB%20(%20hiv%20OR%20aids%20)&sid=c1b2ba28-c406-4dc5-9c9f-26ecbd670bcf%40sessionmgr112&vid=5)

#### Limiters

Published Date from: 20030101-20131031

[AB "community based" AND AB Intervention AND AB ( hiv OR aids )](http://web.ebscohost.com/ehost/breadbox/search?term=AB%20%22community%20based%22%20AND%20AB%20Intervention%20AND%20AB%20(%20hiv%20OR%20aids%20)&sid=c1b2ba28-c406-4dc5-9c9f-26ecbd670bcf%40sessionmgr112&vid=7)

#### Limiters

Published Date from: 20030101-20131031

[AB "community activit*" AND AB Intervention AND AB ( hiv OR aids )](http://web.ebscohost.com/ehost/breadbox/search?term=AB%20%22community%20activit*%22%20AND%20AB%20Intervention%20AND%20AB%20(%20hiv%20OR%20aids%20)&sid=c1b2ba28-c406-4dc5-9c9f-26ecbd670bcf%40sessionmgr112&vid=9)

#### Limiters

Published Date from: 20030101-20131031

[AB "community development" AND AB Intervention AND AB ( hiv OR aids )](http://web.ebscohost.com/ehost/breadbox/search?term=AB%20%22community%20development%22%20AND%20AB%20Intervention%20AND%20AB%20(%20hiv%20OR%20aids%20)&sid=c1b2ba28-c406-4dc5-9c9f-26ecbd670bcf%40sessionmgr112&vid=11)

#### Limiters

Published Date from: 20030101-20131031

AB "capacity building" AND AB Intervention AND AB ( hiv OR aids ) AND AB Community

#### Limiters

Published Date from: 20030101-20131031

**PsycINFO**

[AB "community mobili*" AND AB ( hiv OR aids )](http://web.ebscohost.com/ehost/breadbox/search?term=AB%20%22community%20mobili*%22%20AND%20AB%20(%20hiv%20OR%20aids%20)&sid=739ce657-2ce8-4ec4-8ae8-d0daed64d1b8%40sessionmgr114&vid=14)

#### Limiters

Published Date from: 20030101-20131031

[AB "community particip*" AND AB ( hiv OR aids )](http://web.ebscohost.com/ehost/breadbox/search?term=AB%20%22community%20particip*%22%20AND%20AB%20(%20hiv%20OR%20aids%20)&sid=739ce657-2ce8-4ec4-8ae8-d0daed64d1b8%40sessionmgr114&vid=19)

#### Limiters

#### Published Date from: 20030101-20131031

[AB "community led" AND AB ( hiv OR aids )](http://web.ebscohost.com/ehost/breadbox/search?term=AB%20%22community%20led%22%20AND%20AB%20(%20hiv%20OR%20aids%20)&sid=739ce657-2ce8-4ec4-8ae8-d0daed64d1b8%40sessionmgr114&vid=8)

#### Limiters

Published Date from: 20030101-20131031

[AB "community based" AND AB Intervention AND AB ( hiv OR aids )](http://web.ebscohost.com/ehost/breadbox/search?term=AB%20%22community%20based%22%20AND%20AB%20Intervention%20AND%20AB%20(%20hiv%20OR%20aids%20)&sid=739ce657-2ce8-4ec4-8ae8-d0daed64d1b8%40sessionmgr114&vid=28)

#### Limiters

Published Date from: 20030101-20131031

Peer Reviewed

#### Source Types

All Journals

[AB "community activit*" AND AB Intervention AND AB ( hiv OR aids )](http://web.ebscohost.com/ehost/breadbox/search?term=AB%20%22community%20activit*%22%20AND%20AB%20Intervention%20AND%20AB%20(%20hiv%20OR%20aids%20)&sid=739ce657-2ce8-4ec4-8ae8-d0daed64d1b8%40sessionmgr114&vid=42)

#### Limiters

Published Date from: 20030101-20131031

[AB "community development" AND AB Intervention AND AB ( hiv OR aids )](http://web.ebscohost.com/ehost/breadbox/search?term=AB%20%22community%20development%22%20AND%20AB%20Intervention%20AND%20AB%20(%20hiv%20OR%20aids%20)&sid=739ce657-2ce8-4ec4-8ae8-d0daed64d1b8%40sessionmgr114&vid=37)

#### Limiters

Published Date from: 20030101-20131031

[AB "capacity building" AND AB Intervention AND AB Community AND AB ( hiv OR aids )](http://web.ebscohost.com/ehost/breadbox/search?term=AB%20%22capacity%20building%22%20AND%20AB%20Intervention%20AND%20AB%20Community%20AND%20AB%20(%20hiv%20OR%20aids%20)&sid=739ce657-2ce8-4ec4-8ae8-d0daed64d1b8%40sessionmgr114&vid=46)

#### Limiters

Published Date from: 20030101-20131031
